# Novel Cemented Technique for Trochanteric Fixation and Reconstruction of the Abductor Mechanism in Proximal and Total Femoral Arthroplasty: An Observational Study

**DOI:** 10.1016/j.artd.2021.06.009

**Published:** 2021-08-09

**Authors:** Brian T. Muffly, Kyle T. Boden, Cale A. Jacobs, Patrick W. O’Donnell, Stephen T. Duncan

**Affiliations:** Department of Orthopaedic Surgery & Sports Medicine, University of Kentucky, Lexington, KY, USA

**Keywords:** Abductor reconstruction, Femoral endoprosthesis, Megaprosthesis, Greater trochanter, Reconstruction

## Abstract

**Background:**

Little evidence exists regarding the clinical outcomes of cemented trochanteric fixation for abductor mechanism reconstruction in proximal or total femoral replacements. Clinical outcomes were assessed for a novel cemented technique for trochanteric fixation in femoral megaprostheses.

**Methods:**

A descriptive series of 13 patients who underwent proximal or total femoral arthroplasty from 2016 to 2019 were reviewed. Radiographic trochanteric displacement >1 cm defined construct failure. A Kaplan-Meier survival analysis was performed to determine survival rates for these cemented constructs*.* Demographic information was obtained to better characterize the patient population in whom this technique was used.

**Results:**

Eleven patients were included (age = 63.6 years; 45.4% females; body mass index = 31.7). Mean time to final radiographic follow-up was 73.8 weeks. Three of 11 (27.2%) patients had construct failure. Overall, survival at 1 year was 81.8%. At 2 years, survival of cemented constructs was 65.5%. More construct failures occurred in patients who sustained a postoperative dislocation than in those who did not (*P* = .05).

**Conclusions:**

This novel cemented trochanteric fixation technique for reconstruction of the abductor mechanism in femoral megaprostheses had 81.8% survival at 1 year postoperatively. While longitudinal comparative studies with larger samples are needed, the cemented technique may provide a viable alternative to traditional cementless methods of trochanteric fixation. Increased construct failure rates after postoperative dislocation highlight the importance of robust abductor reconstruction in these implants.

## Introduction

Proximal and total femoral replacements are used to address complex femoral bone abnormalities. These megaprostheses have long been used in the setting of extensive bone loss related to musculoskeletal neoplastic disease [1]. More recently, modular proximal femoral replacements have been found to be viable options in the primary and revision fracture settings [2], as well as in revision total hip arthroplasty and periprosthetic fracture when there is massive proximal femoral bone loss [[3], [4], [5]]. In appropriately selected patients, significant improvements in functional outcomes and quality of life are seen after the use of proximal femoral replacements [6,7].

One of the most problematic aspects of proximal and total femoral megaprostheses is the loss of proximal soft-tissue attachments. The importance of re-establishing the abductor mechanism for prosthesis stability and good functional outcome has been demonstrated in both total hip arthroplasty as well as in reconstruction using proximal/total femoral megaprostheses [8,9]. Inability to reattach the abductors can cause painful Trendelenburg gait, and the inappropriate reconstruction of the abductor mechanism is also believed to be an important cause of instability leading to dislocation [9]. The incidence of dislocation in the literature after proximal and total femoral arthroplasty is as high as 9.5%-13% [4,5,10]. Ogilvie et al. demonstrated a trend toward less disability in patients with abductor soft-tissue repair compared to patients without abductor repair [11].

While the need for abductor reconstruction is clear, little is known regarding the ideal technique for doing so. Biomechanical studies suggest that an abductor muscle insertion located superiorly and laterally on the proximal femoral megaprostheses will optimize abductor moment in single-limb stance [12]. The use of trochanteric claw plate in complex revision hip arthroplasty significantly improves final hip stability compared with wire fixation of the trochanter alone [13]. Studies have also demonstrated varying functional results when comparing direct repair of the trochanter to the prosthesis with abductor soft-tissue repair [11,14]. Experience at our institution has seen failures of trochanteric fixation (using combinations of cable fixation ± trochanteric plate, or suture alone) and subsequent hardware migration in the setting of these megaprostheses. Drawing from the experience seen with allograft-prosthetic composite fixation for proximal femoral bone reconstruction, the use of cemented fixation has not been described for trochanteric fixation with the use of megaprostheses. Little evidence exists regarding the clinical outcomes of cemented trochanteric fixation for abductor mechanism reconstruction in proximal or total femoral replacements. Our aim was to identify those patients who have undergone proximal or total femoral replacement and determine the clinical outcomes after the use of a novel cemented technique for trochanteric fixation.

## Material and methods

We performed an institutional review board–approved retrospective review of 13 patients who underwent either proximal or total femoral replacement between November 2016 and December 2019 at a single institution. Procedures were performed by 4 surgeons (2 adult reconstruction specialists, one musculoskeletal oncologist, and one traumatologist). Trochanteric fragment fixation in proximal and total femoral replacements was performed using a cemented fixation construct. Beginning in November 2016, after a cementless construct failure with migration of the trochanteric plate to the popliteal fossa that necessitated urgent removal, surgeons at our institution began using a cemented construct in these procedures. Anteroposterior and lateral radiographs were routinely obtained in the recovery room and were repeated at subsequent follow-up visits based on individual surgeon preference. All patients had radiographic follow-up of at least 4 weeks (range, 5.7-144.8 weeks). Patients who underwent cementless methods of trochanteric fixation (using combinations of cable fixation ± trochanteric plate, or suture alone; n = 2) were excluded. Eleven patients were included (Table 1).

**Figure 1:**
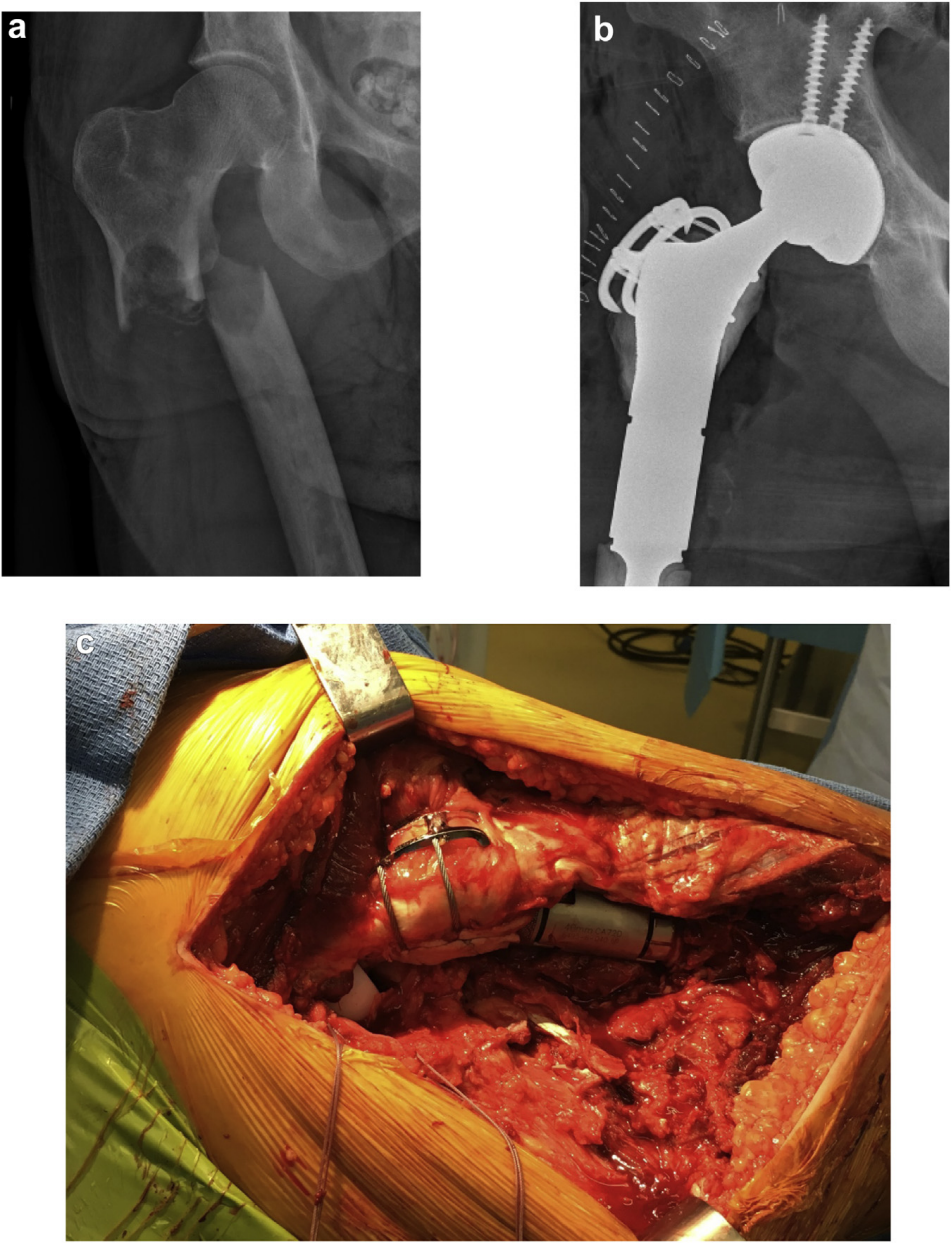
Preoperative (a) and immediate postoperative (b) radiographs. Intraoperative photograph at completion of cemented trochanteric fixation technique in a proximal femoral replacement (c).

All 11 cemented trochanteric fixation constructs were the same. The technique occurred via the use of a trochanteric slide osteotomy with preservation of the abductor muscle insertions and vastus sleeve to the residual greater trochanter. The prosthesis was placed using a standard posterior approach, with distal extension as needed based on surgeon preference. Bearing type was selected based on surgeon preference and stability at the time of surgery. After both the femoral head and final implant were in place, a cable passer was introduced posterior to the femoral implant at the level of the proximal, porous portion of the implant. Cables were passed to encompass the osteotomized trochanteric fragment and the femoral implant. The surgeon then selected a trochanteric plate for the osteotomized fragment and partially tightened the cables to ensure appropriate plate sizing and positioning. The trochanteric plate was impacted to mark the desired position of the plate. Cables were loosened, and the trochanteric fragment was everted. The recipient site at the proximal aspect of the implant was dried, and cement was applied to this area. Cement was molded around the lateral prosthesis and into the proximal porous coating to enhance fixation. Additional cement was applied to the undersurface of the osteotomized trochanteric fragment. The trochanteric piece was reduced back down, and the plate replaced at the site of previous impacted markings. The construct was impacted into place while an assistant held tension on the trochanter distally. Additional cement was placed posterolaterally at the trochanter-implant interface and was interdigitated with the cables. Tensioners were applied, and the cables were tightened according to the manufacturer's instructions. A dry lap was used to dry the area where cement has been applied and to further mold the cement over the cables. After the cement cured, cables were crimped and cut (Fig. 1). The hip was taken through a range of motion to ensure hip stability, and there was no construct impingement. Technique video may be found here (https://www.youtube.com/watch?v=-UqDF2ENjas). Patients were maintained on posterior hip precautions for 6 weeks in addition to no active hip abduction. Abduction pillows were used when patients were in the supine position. Routine hip abduction bracing was not used [15].

**Figure 2:**
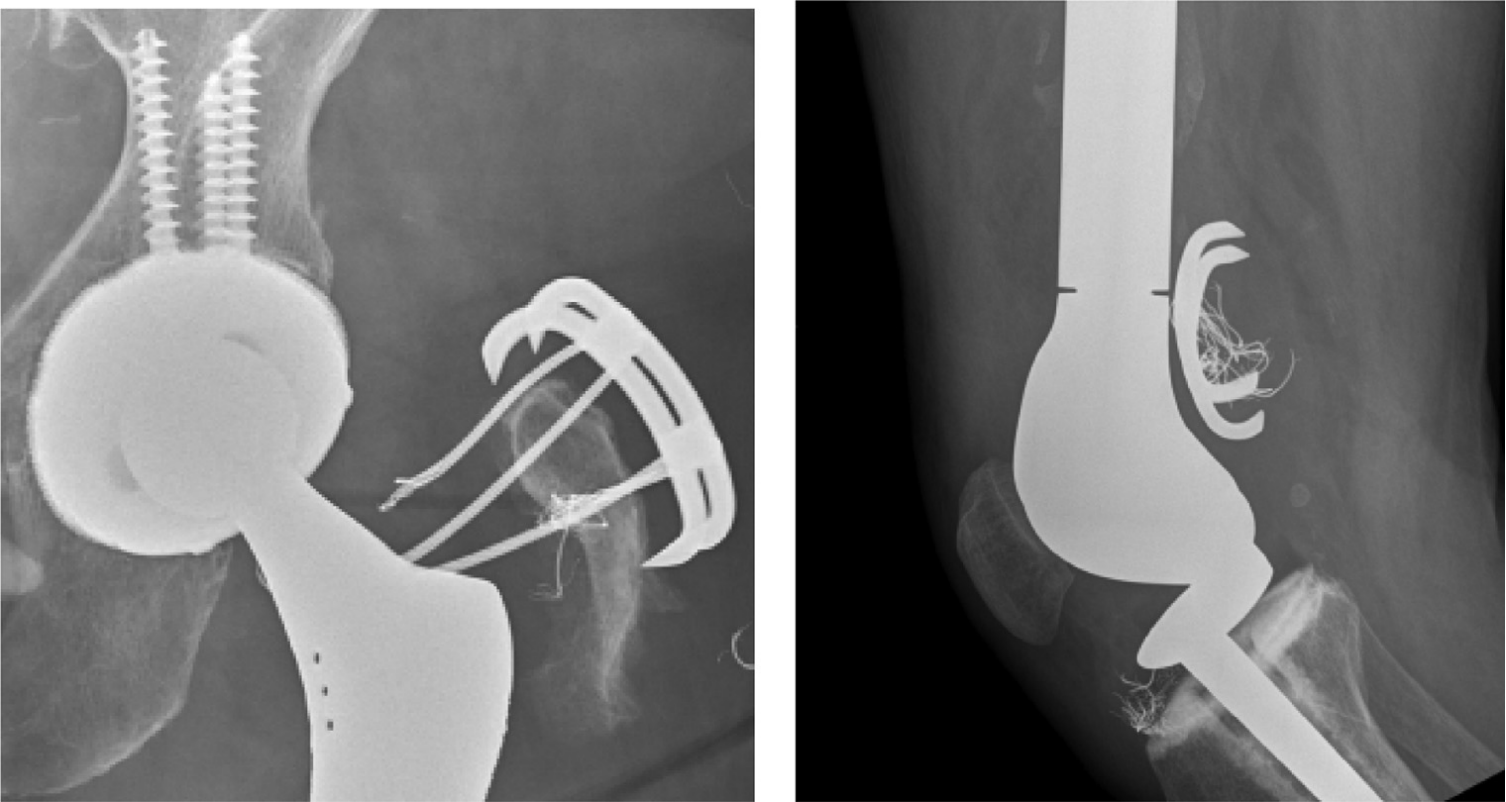
Examples of trochanteric fixation construct failure in 2 separate patients. These 2 failures are from patients in which cementless trochanteric fixation was utilized (prior to the described technique of cemented fixation at our institution).

Radiographic analysis was performed electronically by 2 independent evaluators (B.T.M., K.T.B.) using the ruler function of the digital radiographic system (McKesson, San Francisco, CA). Evaluators were not the operating surgeon. Assessment of patient radiographs in comparison to immediate postoperative films for timing of construct failure was performed (Fig. 2). Failure was defined as trochanteric displacement >1 cm [16,17]. The diameter of the femoral head was measured on each film and was used to correct for differences in magnification. Any disputes between the independent evaluators were resolved by the senior author, a fellowship-trained arthroplasty surgeon (intraclass correlation coefficient [ICC] > 0.90 for interobserver and intraobserver reliability). Patient, surgical, and follow-up data were collected from the electronic medical record. Basic demographic data, need for reoperation, dislocation episodes, and final ambulatory status were recorded. A Kaplan-Meier analysis was performed to determine survival of cemented constructs. All analyses were performed using SPSS Statistics 26 (IBM, Armonk, NY).

## Results

Eleven patients met inclusion criteria and were analyzed. Mean time to final radiographic and clinical follow-up for all patients was 73.8 weeks and 67.3 weeks, respectively. Five patients underwent placement of megaprosthesis for tumor/pathologic fracture, 3 for periprosthetic fracture, 2 for osteolysis/aseptic loosening, and 1 for nonunion.

**Figure 3:**
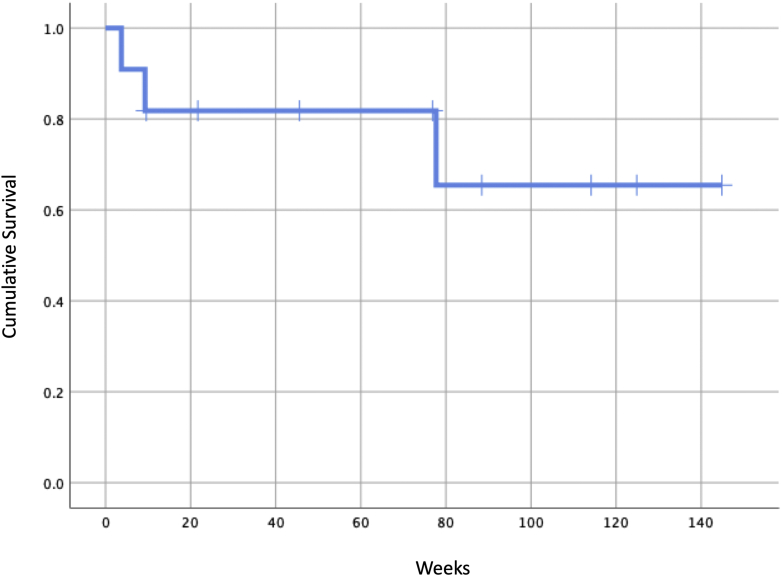
Kaplan-Meier survival curve for cemented constructs.

Cemented construct failure was seen in 3 of 11 patients (27.2%). Radiographic failure of cemented constructs was seen at an average of 30.2 ± 41.2 weeks. Overall, survival of cemented constructs at approximately 1 year was 81.8%. Survival at 2 years was 65.5% (Fig. 3). At the time of writing this article, however, only 4 of 11 patients (36.3%) had follow-up beyond 2 years.

There were 7 total reoperations among 3 patients (27.2%). Four of 7 (57.1%) reoperations were for recurrent instability/dislocation, one for infection (14.2%), one for removal of hardware (14.2%), and one for evacuation of sterile, postoperative hematoma (14.2%). Two patients underwent multiple reoperations. Clinically, 10 patients needed cane/walker for ambulatory assistance at final clinical follow-up. One patient did not require any ambulatory aid, and no patient needed a wheelchair.

A total of 1 bipolar femoral head, 4 modular dual mobility, and 6 constrained bearings were used. Overall, 2 of 11 (18.1%) patients had a dislocation event postoperatively (1 modular dual mobility, 1 constrained liner). Despite small sample sizes, in an exploratory comparison, there was a significantly greater number of construct failures for those patients who sustained a postoperative dislocation than for those without a dislocation event (2/2, 100% vs 1/9, 11.1%, *P* = .05). Both the patients with postoperative dislocation required reoperation for recurrent dislocation and instability.

## Discussion

We present the early clinical results for our initial series of patients treated with a novel cemented construct for reconstruction of the abductor mechanism in proximal and total femoral arthroplasty. These cemented constructs had 81.8% survival at 1 year and 65.5% at 2 years after surgery. Larger, comparative longitudinal studies are necessary to determine if clinical performance of the cemented technique makes it a viable alternative to conventional cementless techniques. Similarly, additional studies are needed to confirm the current finding that patients who sustained a dislocation event postoperatively were significantly more likely to have failure of the cemented abductor mechanism reconstruction. This novel cemented fixation strategy is the authors’ preferred technique of trochanteric fixation in femoral megaprostheses. Furthermore, the significantly increased rate of construct failure after postoperative dislocation continues to highlight the importance of robust abductor mechanism reconstruction in the setting of these implants. Although the authors are unable to say whether dislocation caused the construct failure or if the construct failure proceeded the dislocation, we speculate the latter to be more plausible.

Before undergoing proximal or total femoral arthroplasty, patients should be educated that the main complications of this procedure are instability and infection. As demonstrated by the 27.2% overall reoperation rate seen in our study, it is not uncommon that additional procedure(s) be needed. As only one of 11 (9.09%) patients was ambulating independently at an average of 67.3 weeks postoperatively, it is also reasonable to counsel that patients will likely need an assistive device for ambulation for at least 1 year postoperatively.

Overall, the 18.1% dislocation rate was slightly higher than that noted in previous studies [4,5,10]. Both our patients with dislocations possessed at least 2 of the risk factors for proximal femoral instability as determined by Henderson et al. (age >60 years, female gender, malignant primary bone tumor, and benign condition, but not metastatic disease or soft-tissue tumors) [12]. Dislocation and infection, in alignment with previous studies, were found to be the primary complications of proximal and total femoral megaprostheses [5,10]. Although no patient used a hip abduction brace postoperatively, revision total hip arthroplasty literature suggests that precautionary mobility restrictions/bracing offer little benefit in protecting the abductor mechanism reconstruction in these megaprostheses [15].

This study has several limitations. Given the relatively rare use of femoral megaprostheses and the single-center nature of this observational study, the sample size for analysis is small. The relatively rare use of these implants, coupled with the heterogeneity of methods for trochanteric fixation, makes “clean” comparisons between techniques difficult (if not impossible). Furthermore, the multiple indications for proximal and total femoral arthroplasty lend itself to a heterogenous study population. In addition, because of the small sample size, we could not statistically adjust for confounders that may have contributed to the survival of the construct such as patient age. Future multicenter efforts are needed to obtain an appropriately powered study that can draw more substantive conclusions regarding both the ideal technique by which the abductor mechanism should be reattached to these implants and the clinical outcomes of a given fixation technique. Second, as a major indication for use of femoral megaprostheses is in the setting of tumor and/or pathologic fracture, many patients undergoing proximal or total femoral arthroplasty have decreased longevity in the setting of their pathology and thus have both decreased radiographic and clinical follow-up. Finally, implementation of the novel technique of cemented trochanteric fixation did not begin at our institution until late 2016. While we believe this novel technique to be straightforward and reproducible, there are fewer cases of and shorter follow-up for those who underwent cemented trochanteric fixation as opposed to other, prior cementless forms of trochanteric fixation at our institution. While initial survivorship at 2 years is promising, longer follow-up is also needed to more accurately assess results at this time point. To our knowledge, this is the first study examining cemented trochanteric fixation constructs in the setting of proximal and total femoral arthroplasty. Future studies may aim to quantify patient-reported pain and functional outcomes after cement fixation and/or perform cadaveric biomechanical studies assessing load-to-failure of the technique.

## Conclusions

Survival of the cemented construct was found to be 81.8% at 1 year postoperatively. While survivorship at 2 years postoperatively is promising at 65.5%, longer follow-up is needed to more accurately produce results at this time point. Those with postoperative dislocation are more likely to have failure of the abductor mechanism repair. Based on this cohort, our institution currently prefers the technique of cemented trochanteric fixation. Given that the use femoral megaprostheses is relatively rare, multicenter studies with larger sample sizes and longer radiographic/clinical follow-up are needed to draw substantive conclusions regarding the ideal technique by which the abductor mechanism should be reattached to these implants as well as the associated clinical and patient-reported outcomes.

**Table 1:** Demographics and timing variables for cemented constructs.

	Cemented constructs
Number	11
Age (y)	63.6 ± 13.1 (42.2-84.8)
Body mass index	31.7 ± 7.1 (22.6-47.6)
Mean time to final radiographic follow-up (wk)	73.8 ± 47.2
Mean time to final clinical follow-up (wk)	67.3 ± 48.4
Mean time to radiographic construct failure (wk)	30.2 ± 41.2
Mean time to first additional surgery (wk)	5.6 ± 3.2

## Conflicts of interest

The authors declare that they have no known competing financial interests or personal relationships that could have appeared to influence the work reported in this article.

## Appendix A Supplementary data

Conflict of Interest Statement for Boden

Conflict of Interest Statement for Jacobs

Conflict of Interest Statement for Duncan

Conflict of Interest Statement for Muffly

Conflict of Interest Statement for O’Donnell
