# Supplementary material for: Novel Cemented Technique for Trochanteric Fixation and Reconstruction of the Abductor Mechanism in Proximal and Total Femoral Arthroplasty: An Observational Study
Source: Arthroplast Today. 2021 Aug 9;11:10–4. doi: 10.1016/j.artd.2021.06.009 (PMC8360973; doi:10.1016/j.artd.2021.06.009)
Supplement: Conflict of Interest Statement for Jacobs [file mmc2.pdf]

INDIVIDUAL CONFLICT OF INTEREST STATEMENT

American Association of Hip and Knee Surgeons

(Adopted from the American Academy of Orthopaedic Surgeons disclosure statement)

The following form **must be filled out completely and submitted by each author (example, 6 authors, 6 forms). All items require a response. If there is no relevant disclosure for a given item, enter "*None*."**

Novel cemented versus conventional cementless technique for trochanteric fixation and reconstruction of the abductor mechanism in proximal and total femoral arthroplasty

Manuscript Title

1. Royalties from a company or supplier (The following conflicts were disclosed): None

2. Speakers bureau/paid presentations for a company or supplier (The following conflicts were disclosed): None

3A. Paid employee for a company or supplier (The following conflicts were disclosed): None

3B. Paid consultant for a company or supplier (The following conflicts were disclosed):

- Flexion Therapeutics

3C. Unpaid consultants for a company or supplier (The following conflicts were disclosed): None

4. Stock or stock options in a company or supplier (The following conflicts were disclosed): None

5. Research support from a company or supplier as a Principal Investigator (The following conflicts were disclosed):

- Bonesupport
- Flexion Therapeutics
- Medtronic
- Smith & Nephew
- Zimmer Biomet

6. Other financial or material support from a company or supplier (The following conflicts were disclosed): None

7. Royalties, financial or material support from publishers (The following conflicts were disclosed): None

8. Medical/Orthopaedic publications editorial/governing board (The following conflicts were disclosed):

- Journal of Sport Rehabilitation

9. Board member/committee appointments for a society (The following conflicts were disclosed): None

Each author must sign AND print or type his/her name, date and submit a separate form

In addition, one BLINDED Conflict of Interest form (no author names used) should be submitted per manuscript with all author disclosures.

Cale A. Jacobs	[signature]	9/27/20
Author Name (Print or Type)	Author Signature	Date
